# Supplementary material for: Can scientists fill the science journalism void? Online public engagement with science stories authored by scientists
Source: PLoS One. 2020 Jan 8;15(1):e0222250. doi: 10.1371/journal.pone.0222250 (PMC6948730; doi:10.1371/journal.pone.0222250)
Supplement: S1 Table — Data of date of publication, number of clicks (views), ‘Likes’, Comments and average time on page is presented for each pair of items (e.g. scientist or reporter) on both news sites. (DOCX) [file pone.0222250.s001.docx]

**S1 Table: paired items datasets with ‘Google analytics’ data.** Data of date of publication, number of clicks (views), ‘Likes’, Comments and average time on page is presented for each pair of items (e.g. scientist or reporter) on both news sites.

| **Pair #** | | **Author** | | **Date** | | **Views** | | **Likes** | | **Comments** | | | **Avg. Time on Page** | |
| --- | --- | --- | --- | --- | --- | --- | --- | --- | --- | --- | --- | --- | --- | --- |
| **Ynet** | |  | |  | |  | |  | |  | |  | | |
| 1 | | Scientist | | 25.01.16 | | 17,350 | | 16 | | 11 | | 0:26:56 | | |
| 1 | | Reporter | | 25.01.16 | | 7,871 | | 8 | | 2 | | 0:22:08 | | |
| 2 | | Scientist | | 04.02.16 | | 68,740 | | 131 | | 114 | | 0:25:29 | | |
| 2 | | Reporter | | 04.02.16 | | 13,669 | | 11 | | 21 | | 0:25:30 | | |
| 3 | | Scientist | | 08.03.16 | | 30,148 | | 0 | | 66 | | 0:23:59 | | |
| 3 | | Reporter | | 07.03.16 | | 5,195 | | 20 | | 14 | | 0:23:12 | | |
| 4 | | Scientist | | 14.03.16 | | 116,913 | | 269 | | 152 | | 0:25:47 | | |
| 4 | | Reporter | | 14.03.16 | | 12,475 | | 0 | | 24 | | 0:24:19 | | |
| 5 | | Scientist | | 27.03.16 | | 21,850 | | 114 | | 28 | | 0:25:08 | | |
| 5 | | Reporter | | 27.03.16 | | 77,671 | | 0 | | 99 | | 0:21:44 | | |
| 6 | | Scientist | | 30.03.16 | | 35,519 | | 127 | | 33 | | 0:25:31 | | |
| 6 | | Reporter | | 30.03.16 | | 22,762 | | 54 | | 74 | | 0:22:23 | | |
| 7 | | Scientist | | 01.04.16 | | 11,724 | | 48 | | 21 | | 0:25:36 | | |
| 7 | | Reporter | | 03.04.16 | | 51,919 | | 0 | | 178 | | 0:25:05 | | |
| 8 | | Scientist | | 19.04.16 | | 14,802 | | 14 | | 13 | | 0:24:23 | | |
| 8 | | Reporter | | 19.04.16 | | 61,481 | | 0 | | 35 | | 0:24:19 | | |
| 9 | | Scientist | | 05.05.16 | | 36,038 | | 118 | | 38 | | 0:27:22 | | |
| 9 | | Reporter | | 06.05.16 | | 23,568 | | 92 | | 48 | | 0:26:50 | | |
| 10 | | Scientist | | 12.05.16 | | 8,892 | | 8 | | 11 | | 0:27:01 | | |
| 10 | | Reporter | | 12.05.16 | | 27,580 | | 44 | | 13 | | 0:24:16 | | |
| 11 | | Scientist | | 16.05.16 | | 9,658 | | 48 | | 66 | | 0:24:47 | | |
| 11 | | Reporter | | 18.05.16 | | 9,653 | | 0 | | 11 | | 0:22:36 | | |
| 12 | | Scientist | | 02.06.16 | | 5,049 | | 44 | | 5 | | 0:26:44 | | |
| 12 | | Reporter | | 02.06.16 | | 67,992 | | 214 | | 77 | | 0:26:17 | | |
| 13 | | Scientist | | 28.06.16 | | 31,222 | | 7 | | 33 | | 0:26:25 | | |
| 13 | | Reporter | | 27.06.16 | | 37,113 | | 0 | | 31 | | 0:28:22 | | |
| 14 | | Scientist | | 02.07.16 | | 9,241 | | 30 | | 7 | | 0:26:52 | | |
| 14 | | Reporter | | 03.07.16 | | 38,460 | | 0 | | 97 | | 0:26:00 | | |
| 15 | | Scientist | | 04.07.16 | | 4,203 | | 6 | | 4 | | 0:18:25 | | |
| 15 | | Reporter | | 04.07.16 | | 8,581 | | 17 | | 5 | | 0:23:20 | | |
| 16 | | Scientist | | 07.07.16 | | 26,388 | | 25 | | 36 | | 0:25:39 | | |
| 16 | | Reporter | | 06.07.16 | | 9,291 | | 0 | | 18 | | 0:24:37 | | |
| 17 | | Scientist | | 20.07.16 | | 48,207 | | 52 | | 42 | | 0:26:28 | | |
| 17 | | Reporter | | 20.07.16 | | 28,302 | | 0 | | 89 | | 0:26:27 | | |
| 18 | | Scientist | | 28.07.16 | | 20,549 | | 22 | | 27 | | 0:21:25 | | |
| 18 | | Reporter | | 27.07.16 | | 12,180 | | 74 | | 19 | | 0:21:12 | | |
| 19 | | Scientist | | 01.08.16 | | 32,223 | | 49 | | 34 | | 0:27:04 | | |
| 19 | | Reporter | | 01.08.16 | | 30,839 | | 0 | | 72 | | 0:24:28 | | |
| 20 | | Scientist | | 02.08.16 | | 25,157 | | 42 | | 53 | | 0:26:16 | | |
| 20 | | Reporter | | 02.08.16 | | 14,162 | | 0 | | 20 | | 0:16:39 | | |
| 21 | | Scientist | | 10.08.16 | | 51,043 | | 336 | | 175 | | 0:22:34 | | |
| 21 | | Reporter | | 10.08.16 | | 17,031 | | 18 | | 21 | | 0:25:04 | | |
| 22 | | Scientist | | 15.08.16 | | 15,442 | | 55 | | 30 | | 0:22:27 | | |
| 22 | | Reporter | | 15.08.16 | | 7,590 | | 0 | | 18 | | 0:21:04 | | |
| 23 | | Scientist | | 21.08.16 | | 61,330 | | 30 | | 26 | | 0:25:39 | | |
| 23 | | Reporter | | 18.08.16 | | 16,268 | | 0 | | 74 | | 0:19:31 | | |
| 24 | | Scientist | | 22.08.16 | | 101,204 | | 45 | | 63 | | 0:25:50 | | |
| 24 | | Reporter | | 22.08.16 | | 10,086 | | 0 | | 0 | | 0:23:41 | | |
| 25 | | Scientist | | 24.08.16 | | 25,352 | | 100 | | 11 | | 0:26:56 | | |
| 25 | | Reporter | | 24.08.16 | | 30,405 | | 79 | | 14 | | 0:26:29 | | |
| 26 | | Scientist | | 24.08.16 | | 198,813 | | 3000 | | 307 | | 0:26:10 | | |
| 26 | | Reporter | | 24.08.16 | | 33,165 | | 0 | | 107 | | 0:24:36 | | |
| 27 | | Scientist | | 29.08.16 | | 5,587 | | 17 | | 9 | | 0:26:56 | | |
| 27 | | Reporter | | 29.08.16 | | 15,170 | | 5 | | 19 | | 0:20:25 | | |
| 28 | | Scientist | | 10.09.15 | |  | | 44 | | 19 | | 0:21:29 | | |
| 28 | | Reporter | | 10.09.15 | | 117,329 | | 120 | | 255 | | 0:25:36 | | |
| 29 | | Scientist | | 22.10.16 | | 17,295 | | 52 | | 130 | | 0:13:20 | | |
| 29 | | Reporter | | 24.10.16 | | 37,250 | | 111 | | 76 | | 0:28:13 | | |
| 30 | | Scientist | | 07/11/2016 | | 21,002 | | n/a | | 70 | | 0:15:20 | | |
| 30 | | Reporter | | 07/11/2016 | | 4,342 | | n/a | | 5 | | 0:03:38 | | |
| 31 | | Scientist | | 05/12/2016 | | 36,422 | | n/a | | 63 | | 0:13:09 | | |
| 31 | | Reporter | | 05/12/2016 | | 54,195 | | n/a | | 53 | | 0:09:56 | | |
| 32 | | Scientist | | 3/1/2017 | | 24,144 | | n/a | | 37 | | 0:06:38 | | |
| 32 | | Reporter | | 3/1/2017 | | 15,360 | | n/a | | 37 | | 0:05:16 | | |
| 33 | | Scientist | | 17/1/2017 | | 36,408 | | n/a | | 74 | | 0:10:01 | | |
| 33 | | Reporter | | 17/1/2017 | | 83,865 | | n/a | | 153 | | 0:10:02 | | |
| 34 | | Scientist | | 2/2/2017 | | 73,626 | | n/a | | 78 | | 0:05:59 | | |
| 34 | | Reporter | | 2/2/2017 | | 49,940 | | n/a | | 75 | | 0:12:52 | | |
| 35 | | Scientist | | 22/2/2017 | | 140,955 | | n/a | | 268 | | 0:12:57 | | |
| 35 | | Reporter | | 22/2/2017 | | 50,313 | | n/a | | 106 | | 0:07:54 | | |
| 36 | | Scientist | | 8/3/2017 | | 45,869 | | n/a | | 90 | | 0:15:35 | | |
| 36 | | Reporter | | 8/3/2017 | | 17,479 | | n/a | | 48 | | 0:10:45 | | |
| 37 | | Scientist | | 24/3/2017 | | 22,139 | | n/a | | 35 | | 0:08:02 | | |
| 37 | | Reporter | | 24/3/2017 | | 108,259 | | n/a | | 94 | | 0:12:37 | | |
| 38 | | Scientist | | 30/3/2017 | | 154,581 | | n/a | | 138 | | 0:10:37 | | |
| 38 | | Reporter | | 30/3/2017 | | 51,312 | | n/a | | 70 | | 0:11:55 | | |
| 39 | | Scientist | | 10/4/2017 | | 182,228 | | n/a | | 403 | | 0:15:09 | | |
| 39 | | Reporter | | 11/4/2017 | | 14,482 | | n/a | | 31 | | 0:11:12 | | |
| 40 | | Scientist | | 14/4/2017 | | 55,254 | | n/a | | 119 | | 0:07:48 | | |
| 40 | | Reporter | | 14/4/2017 | | 91,328 | | n/a | | 128 | | 0:07:48 | | |
| 41 | | Scientist | | 8/5/2017 | | 55,240 | | n/a | | 18 | | 0:07:48 | | |
| 41 | | Reporter | | 9/5/2017 | | 2,833 | | n/a | | 14 | | 0:01:57 | | |
| 42 | | Scientist | | 27/5/2017 | | 11,185 | | n/a | | 56 | | 0:15:29 | | |
| 42 | | Reporter | | 27/5/2017 | | 19,580 | | n/a | | 52 | | 0:10:30 | | |
| 43 | | Scientist | | 7/6/2017 | | 118,424 | | n/a | | 254 | | 0:14:18 | | |
| 43 | | Reporter | | 7/6/2017 | | 87,276 | | n/a | | 81 | | 0:09:48 | | |
| 44 | | Scientist | | 23/6/2017 | | 52,469 | | n/a | | 153 | | 0:14:49 | | |
| 44 | | Reporter | | 23/6/2017 | | 66,394 | | n/a | | 58 | | 0:11:45 | | |
| 45 | | Scientist | | 21/7/2017 | | 42,701 | | n/a | | 28 | | 0:07:45 | | |
| 45 | | Reporter | | 21/7/2017 | | 16,562 | | n/a | | 41 | | 0:06:48 | | |
| 46 | | Scientist | | 28/7/2017 | | 76,789 | | n/a | | 88 | | 0:07:17 | | |
| 46 | | Reporter | | 28/7/2017 | | 33,089 | | n/a | | 23 | | 0:08:02 | | |
| 47 | | Scientist | | 17/07/2017 | | 23,673 | | n/a | | 43 | | 0:07:06 | | |
| 47 | | Reporter | | 18/07/2017 | | 149,142 | | n/a | | 85 | | 0:08:14 | | |
| 48 | | Scientist | | 19/8/2017 | | 51,961 | | n/a | | 147 | | 0:10:10 | | |
| 48 | | Reporter | | 20/8/2017 | | 23,334 | | n/a | | 37 | | 0:09:27 | | |
| 49 | | Scientist | | 28/8/2017 | | 74,987 | | n/a | | 77 | | 0:08:04 | | |
| 49 | | Reporter | | 28/8/2017 | | 35,716 | | n/a | | 45 | | 0:07:30 | | |
| 50 | | Scientist | | 08/09/2017 | | 18,167 | | n/a | | 10 | | 0:09:01 | | |
| 50 | | Reporter | | 08/09/2017 | | 50,453 | | n/a | | 77 | | 0:09:08 | | |
| 51 | | Scientist | | 15/09/2017 | | 25,467 | | n/a | | 19 | | 0:08:56 | | |
| 51 | | Reporter | | 15/09/2017 | | 51,393 | | n/a | | 94 | | 0:09:57 | | |
| 52 | | Scientist | | 26/11/2017 | | 18,907 | | n/a | | 29 | | 0:08:03 | | |
| 52 | | Reporter | | 28/11/2017 | | 10,085 | | n/a | | 14 | | 0:08:16 | | |
| 53 | | Scientist | | 13/12/2017 | | 269,802 | | n/a | | 258 | | 0:10:43 | | |
| 53 | | Reporter | | 13/12/2017 | | 5,324 | | n/a | | 2 | | 0:08:18 | | |
| 54 | | Scientist | | 22/01/2018 | | 53,359 | | n/a | | 79 | | 0:07:09 | | |
| 54 | | Reporter | | 22/01/2018 | | 94,802 | | n/a | | 65 | | 0:10:26 | | |
| **Mako** |  | |  | |  | |  | |  | |  | | |  |
| 1 | Scientist | | 17/07/2015 | | 27,740 | | 1400 | | 24 | | 0:01:14 | | |  |
| 1 | Reporter | | 21/07/2015 | | 47212 | | 6 | | 18 | | 0:00:40 | | |  |
| 2 | Scientist | | 29/07/2015 | | 22,346 | | 462 | | 45 | | 0:01:37 | | |  |
| 2 | Reporter | | 29/07/2015 | | 7,037 | | 20 | | 1 | | 0:03:18 | | |  |
| 3 | Scientist | | 30/07/2015 | | 50,214 | | 14 | | 8 | | 0:04:48 | | |  |
| 3 | Reporter | | 30/07/2015 | | 95768 | | 38 | | 21 | | 0:07:19 | | |  |
| 4 | Scientist | | 19/08/2015 | | 3,191 | | 141 | | 2 | | 0:02:23 | | |  |
| 4 | Reporter | | 18/08/2015 | | 35499 | | 173 | | 8 | | 0:01:26 | | |  |
| 5 | Scientist | | 24/08/2015 | | 16,908 | | 500 | | 9 | | 0:02:21 | | |  |
| 5 | Reporter | | 23/08/2015 | | 7739 | | 30 | | 5 | | 0:01:31 | | |  |
| 6 | Scientist | | 03/09/2015 | | 75,414 | | 488 | | 7 | | 0:03:05 | | |  |
| 6 | Reporter | | 03/09/2015 | | 21159 | | 42 | | 4 | | 0:01:53 | | |  |
| 7 | Scientist | | 01/10/2015 | | 44,858 | | 34 | | 14 | | 0:04:54 | | |  |
| 7 | Reporter | | 01/10/2015 | | 199033 | | 96 | | 27 | | 0:03:57 | | |  |
| 8 | Scientist | | 08/10/2015 | | 8,658 | | 80 | | 0 | | 0:03:30 | | |  |
| 8 | Reporter | | 11/10/2015 | | 74,193 | | 1K | | 24 | | 0:01:07 | | |  |
| 9 | Scientist | | 29/10/2015 | | 34,996 | | 31 | | 28 | | 0:05:47 | | |  |
| 9 | Reporter | | 29/10/2015 | | 31797 | | 63 | | 8 | | 0:04:37 | | |  |
| 10 | Scientist | | 04/11/2015 | | 6477 | | 0 | | 10 | | 0:01:12 | | |  |
| 10 | Reporter | | 03/11/2015 | | 47001 | | 1 | | 12 | | 0:01:05 | | |  |
| 11 | Scientist | | 05/11/2015 | | 18,637 | | 68 | | 6 | | 0:05:29 | | |  |
| 11 | Reporter | | 05/11/2015 | | 26834 | | 48 | | 15 | | 0:06:48 | | |  |
| 12 | Scientist | | 08/11/2015 | | 33,943 | | 418 | | 22 | | 0:00:40 | | |  |
| 12 | Reporter | | 08/11/2015 | | 7304 | | 304 | | 7 | | 0:01:03 | | |  |
| 13 | Scientist | | 13/11/2015 | | 13,080 | | 3 | | 3 | | 0:02:32 | | |  |
| 13 | Reporter | | 12/11/2015 | | 25400 | | 231 | | 0 | | 0:01:17 | | |  |
| 14 | Scientist | | 22/11/2015 | | 16,575 | | 52 | | 6 | | 0:00:54 | | |  |
| 14 | Reporter | | 23/11/2015 | | 4,413 | | 9 | | 4 | | 0:00:35 | | |  |
| 15 | Scientist | | 08/12/2015 | | 13,161 | | 103 | | 4 | | 0:02:33 | | |  |
| 15 | Reporter | | 08/12/2015 | | 7827 | | 6 | | 9 | | 0:03:24 | | |  |
| 16 | Scientist | | 14/12/2015 | | 10,634 | | 117 | | 4 | | 0:01:29 | | |  |
| 16 | Reporter | | 14/12/2015 | | 9,084 | | 28 | | 0 | | 0:02:47 | | |  |
| 17 | Scientist | | 20/12/2015 | | 6,151 | | 37 | | 5 | | 0:01:12 | | |  |
| 17 | Reporter | | 23/12/2015 | | 8,598 | | 13 | | 7 | | 0:01:16 | | |  |
| 18 | Scientist | | 27/12/2015 | | 13,695 | | 140 | | 1 | | 0:01:10 | | |  |
| 18 | Reporter | | 30/12/2015 | | 52,727 | | 48 | | 11 | | 0:00:55 | | |  |
| 19 | Scientist | | 27/12/2015 | | 13693 | | 140 | | 1 | | 0:01:03 | | |  |
| 19 | Reporter | | 24/12/2015 | | 18880 | | 120 | | 4 | | 0:00:48 | | |  |
| 20 | Scientist | | 01/01/2016 | | 30,335 | | 236 | | 11 | | 0:03:55 | | |  |
| 20 | Reporter | | 01/01/2016 | | 5592 | | 0 | | 1 | | 0:00:40 | | |  |
| 21 | Scientist | | 05/01/2016 | | 7884 | | 9 | | 3 | | 0:01:21 | | |  |
| 21 | Reporter | | 05/01/2016 | | 11658 | | 103 | | 13 | | 0:01:06 | | |  |
| 22 | Scientist | | 07/01/2016 | | 2911 | | 29 | | 2 | | 0:03:12 | | |  |
| 22 | Reporter | | 07/01/2016 | | 41197 | | 79 | | 40 | | 0:02:22 | | |  |
| 23 | Scientist | | 11/01/2016 | | 71,354 | | 159 | | 13 | | 0:03:05 | | |  |
| 23 | Reporter | | 12/01/2016 | | 8,290 | | 24 | | 7 | | 0:04:58 | | |  |
| 24 | Scientist | | 21/01/2016 | | 3,788 | | 1,000 | | 30 | | 0:02:35 | | |  |
| 24 | Reporter | | 20/01/2016 | | 40,999 | | 1,300 | | 93 | | 0:05:03 | | |  |
| 25 | Scientist | | 26/01/2016 | | 10367 | | 8 | | 5 | | 0:01:34 | | |  |
| 25 | Reporter | | 24/01/2016 | | 34793 | | 180 | | 7 | | 0:02:03 | | |  |
| 26 | Scientist | | 02/02/2016 | | 13,986 | | 23 | | 12 | | 0:04:14 | | |  |
| 26 | Reporter | | 02/02/2016 | | 12,732 | | 172 | | 6 | | 0:03:54 | | |  |
| 27 | Scientist | | 14/02/2016 | | 29642 | | 321 | | 7 | | 0:00:58 | | |  |
| 27 | Reporter | | 14/02/2016 | | 7425 | | 34 | | 2 | | 0:01:20 | | |  |
| 28 | Scientist | | 19/02/2016 | | 5,819 | | 9 | | 2 | | 0:03:05 | | |  |
| 28 | Reporter | | 18/02/2016 | | 14,068 | | 1,100 | | 57 | | 0:05:52 | | |  |
| 29 | Scientist | | 23/02/2016 | | 10,535 | | 26 | | 6 | | 0:06:19 | | |  |
| 29 | Reporter | | 23/02/2016 | | 32,316 | | 2,500 | | 72 | | 0:04:47 | | |  |
| 30 | Scientist | | 06/03/2016 | | 8,196 | | 25 | | 4 | | 0:05:00 | | |  |
| 30 | Reporter | | 06/03/2016 | | 4,907 | | 6 | | 7 | | 0:04:54 | | |  |
| 31 | Scientist | | 07/03/2016 | | 10,537 | | 103 | | 6 | | 0:03:37 | | |  |
| 31 | Reporter | | 07/03/2016 | | 39491 | | 587 | | 7 | | 0:01:10 | | |  |
| 32 | Scientist | | 08/03/2016 | | 6,989 | | 36 | | 0 | | 0:03:22 | | |  |
| 32 | Reporter | | 10/03/2016 | | 22249 | | 38 | | 3 | | 0:03:35 | | |  |
| 33 | Scientist | | 20/03/2016 | | 5,311 | | 28 | | 14 | | 0:05:03 | | |  |
| 33 | Reporter | | 21/03/2016 | | 26,417 | | 64 | | 4 | | 0:02:58 | | |  |
| 34 | Scientist | | 22/03/2016 | | 2,526 | | 24 | | 7 | | 0:01:19 | | |  |
| 34 | Reporter | | 23/03/2016 | | 1,817 | | 0 | | 2 | | 0:06:10 | | |  |
| 35 | Scientist | | 01/04/2016 | | 16,442 | | 343 | | 1 | | 0:05:19 | | |  |
| 35 | Reporter | | 30/03/2016 | | 11879 | | 12 | | 1 | | 0:03:23 | | |  |
| 36 | Scientist | | 03/04/2016 | | 9111 | | 24 | | 5 | | 0:00:56 | | |  |
| 36 | Reporter | | 03/04/2016 | | 4711 | | 67 | | 7 | | 0:01:28 | | |  |
| 37 | Scientist | | 03/04/2016 | | 17,375 | | 38 | | 5 | | 0:04:55 | | |  |
| 37 | Reporter | | 03/04/2016 | | 44,926 | | 254 | | 34 | | 0:05:42 | | |  |
| 38 | Scientist | | 13/04/2016 | | 19,958 | | 272 | | 7 | | 0:04:04 | | |  |
| 38 | Reporter | | 15/04/2016 | | 14,431 | | 366 | | 9 | | 0:03:57 | | |  |
| 39 | Scientist | | 25/04/2016 | | 31,538 | | 87 | | 6 | | 0:02:33 | | |  |
| 39 | Reporter | | 25/04/2016 | | 8,451 | | 15 | | 5 | | 0:01:56 | | |  |
| 40 | Scientist | | 03/05/2016 | | 15,434 | | 12 | | 6 | | 0:03:49 | | |  |
| 40 | Reporter | | 04/05/2016 | | 41,259 | | 416 | | 17 | | 0:03:53 | | |  |
| 41 | Scientist | | 05/06/2016 | | 26,594 | | 156 | | 54 | | 0:03:48 | | |  |
| 41 | Reporter | | 06/06/2016 | | 12,350 | | 72 | | 8 | | 0:03:52 | | |  |
| 42 | Scientist | | 07/06/2016 | | 14,260 | | 90 | | 1 | | 0:04:44 | | |  |
| 42 | Reporter | | 07/06/2016 | | 32,893 | | 21 | | 7 | | 0:04:04 | | |  |
| 43 | Scientist | | 09/06/2016 | | 6,539 | | 25 | | 8 | | 0:03:38 | | |  |
| 43 | Reporter | | 09/06/2016 | | 14,380 | | 29 | | 2 | | 0:02:42 | | |  |
| 44 | Scientist | | 10/07/2016 | | 7,261 | | 8 | | 0 | | 0:01:45 | | |  |
| 44 | Reporter | | 10/07/2016 | | 28,184 | | 72 | | 8 | | 0:03:26 | | |  |
| 45 | Scientist | | 12/07/2016 | | 15,702 | | 4 | | 7 | | 0:02:57 | | |  |
| 45 | Reporter | | 12/07/2016 | | 70,295 | | 915 | | 18 | | 0:04:23 | | |  |
| 46 | Scientist | | 20/07/2016 | | 5,545 | | 11 | | 6 | | 0:01:14 | | |  |
| 46 | Reporter | | 20/07/2016 | | 6,997 | | 24 | | 1 | | 0:02:06 | | |  |
| 47 | Scientist | | 25/07/2016 | | 20,830 | | 99 | | 5 | | 0:04:40 | | |  |
| 47 | Reporter | | 28/07/2016 | | 4,592 | | 2 | | 0 | | 0:00:50 | | |  |
| 48 | Scientist | | 03/08/2016 | | 2,994 | | 43 | | 8 | | 0:00:13 | | |  |
| 48 | Reporter | | 03/08/2016 | | 16,737 | | 53 | | 5 | | 0:00:38 | | |  |
| 49 | Scientist | | 07/08/2016 | | 7,967 | | 4 | | 9 | | 0:03:14 | | |  |
| 49 | Reporter | | 08/08/2016 | | 9,800 | | 1 | | 3 | | 0:04:43 | | |  |
| 50 | Scientist | | 11/08/2016 | | 8,733 | | 94 | | 6 | | 0:04:00 | | |  |
| 50 | Reporter | | 09/08/2016 | | 21,232 | | 158 | | 35 | | 0:04:08 | | |  |
| 51 | Scientist | | 15/08/2016 | | 4,594 | | 30 | | 3 | | 0:01:24 | | |  |
| 51 | Reporter | | 15/08/2016 | | 2,490 | | 8 | | 0 | | 0:01:18 | | |  |
| 52 | Scientist | | 18/08/2016 | | 11,565 | | 1,800 | | 1 | | 0:04:25 | | |  |
| 52 | Reporter | | 17/08/2016 | | 25,867 | | 58 | | 4 | | 0:02:38 | | |  |
| 53 | Scientist | | 22/08/2016 | | 854 | | 6 | | 0 | | 0:00:17 | | |  |
| 53 | Reporter | | 22/08/2016 | | 11,052 | | 17 | | 3 | | 0:01:28 | | |  |
| 54 | Scientist | | 01/11/2016 | | 5,698 | | 54 | | 4 | | 0:01:05 | | |  |
| 54 | Reporter | | 03/11/2016 | | 17,302 | | 1,000 | | 26 | | 0:05:27 | | |  |
| 55 | Scientist | | 09/11/2016 | | 9,135 | | 5 | | 0 | | 0:05:18 | | |  |
| 55 | Reporter | | 08/11/2016 | | 11,305 | | 55 | | 13 | | 0:03:43 | | |  |
| 56 | Scientist | | 16/11/2016 | | 11,223 | | 9 | | 1 | | 0:02:55 | | |  |
| 56 | Reporter | | 18/11/2016 | | 2,559 | | 4 | | 3 | | 0:00:46 | | |  |
| 57 | Scientist | | 17/11/2016 | | 4,823 | | 9 | | 2 | | 0:05:08 | | |  |
| 57 | Reporter | | 16/11/2016 | | 4,605 | | 96 | | 11 | | 0:03:26 | | |  |
| 58 | Scientist | | 04/12/2016 | | 1,487 | | 10 | | 1 | | 0:01:42 | | |  |
| 58 | Reporter | | 04/12/2016 | | 7,167 | | 15 | | 2 | | 0:03:48 | | |  |
| 59 | Scientist | | 11/12/2016 | | 14,653 | | 17 | | 9 | | 0:02:53 | | |  |
| 59 | Reporter | | 11/12/2016 | | 12,187 | | 2,100 | | 12 | | 0:01:25 | | |  |
| 60 | Scientist | | 13/12/2016 | | 3,765 | | 291 | | 17 | | 0:03:14 | | |  |
| 60 | Reporter | | 13/12/2016 | | 3,941 | | 6 | | 8 | | 0:05:29 | | |  |
| 61 | Scientist | | 22/12/2016 | | 4,317 | | 45 | | 4 | | 0:02:33 | | |  |
| 61 | Reporter | | 22/12/2016 | | 2,591 | | 47 | | 4 | | 0:03:52 | | |  |
| 62 | Scientist | | 30/12/2016 | | 5,961 | | 57 | | 2 | | 0:03:24 | | |  |
| 62 | Reporter | | 2/1/2017 | | 7,384 | | 131 | | 6 | | 0:02:50 | | |  |
| 63 | Scientist | | 1/1/2017 | | 651 | | 0 | | 0 | | 0:00:00 | | |  |
| 63 | Reporter | | 3/1/2017 | | 686 | | 10 | | 1 | | 0:01:20 | | |  |
| 64 | Scientist | | 19/1/2017 | | 5,192 | | 16 | | 6 | | 0:04:52 | | |  |
| 64 | Reporter | | 18/1/2017 | | 3,867 | | 10 | | 3 | | 0:01:57 | | |  |
| 65 | Scientist | | 22/02/2017 | | 1,294 | | 6 | | 1 | | 0:02:01 | | |  |
| 65 | Reporter | | 20/2/2017 | | 4,353 | | 0 | | 1 | | 0:02:07 | | |  |
| 66 | Scientist | | 12/3/2017 | | 20,315 | | 305 | | 11 | | 0:04:36 | | |  |
| 66 | Reporter | | 15/3/2017 | | 103,763 | | 433 | | 12 | | 0:01:37 | | |  |
| 67 | Scientist | | 14/3/2017 | | 20,351 | | 269 | | 13 | | 0:03:03 | | |  |
| 67 | Reporter | | 14/3/2017 | | 3,215 | | 6 | | 0 | | 0:01:33 | | |  |
| 68 | Scientist | | 20/3/2017 | | 3,792 | | 17 | | 2 | | 0:03:20 | | |  |
| 68 | Reporter | | 20/3/2017 | | 8,144 | | 8 | | 0 | | 0:03:35 | | |  |
| 69 | Scientist | | 21/3/2017 | | 3,902 | | 29 | | 1 | | 0:01:02 | | |  |
| 69 | Reporter | | 23/3/2017 | | 4,175 | | 10 | | 7 | | 0:01:28 | | |  |
| 70 | Scientist | | 29/3/2017 | | 2,891 | | 2 | | 0 | | 0:03:11 | | |  |
| 70 | Reporter | | 28/3/2017 | | 4,776 | | 2 | | 5 | | 0:02:38 | | |  |
| 71 | Scientist | | 3/4/2017 | | 2,463 | | 6 | | 2 | | 0:03:53 | | |  |
| 71 | Reporter | | 2/4/2017 | | 4,241 | | 4 | | 4 | | 0:02:53 | | |  |
| 72 | Scientist | | 5/4/2017 | | 10,507 | | 3 | | 0 | | 0:01:19 | | |  |
| 72 | Reporter | | 5/4/2017 | | 28,262 | | 84 | | 3 | | 0:02:54 | | |  |
| 73 | Scientist | | 6/4/2017 | | 2,968 | | 18 | | 0 | | 0:03:12 | | |  |
| 73 | Reporter | | 4/4/2017 | | 7,007 | | 17 | | 5 | | 0:02:05 | | |  |
| 74 | Scientist | | 16/4/2017 | | 6,742 | | 4 | | 3 | | 0:03:26 | | |  |
| 74 | Reporter | | 16/4/2017 | | 5,138 | | 145 | | 0 | | 0:02:56 | | |  |
| 75 | Scientist | | 19/4/2017 | | 12,014 | | 140 | | 13 | | 0:04:42 | | |  |
| 75 | Reporter | | 18/4/2017 | | 5,461 | | 88 | | 4 | | 0:00:14 | | |  |
| 76 | Scientist | | 24/4/2017 | | 7,273 | | 3 | | 2 | | 0:03:00 | | |  |
| 76 | Reporter | | 24/4/2017 | | 34,084 | | 666 | | 15 | | 0:03:41 | | |  |
| 77 | Scientist | | 26/4/2017 | | 9,055 | | 20 | | 4 | | 0:00:54 | | |  |
| 77 | Reporter | | 23/4/2017 | | 4,842 | | 2 | | 1 | | 0:00:56 | | |  |
| 78 | Scientist | | 8/5/2017 | | 6,918 | | 6 | | 4 | | 0:01:03 | | |  |
| 78 | Reporter | | 9/5/2017 | | 13,433 | | 582 | | 8 | | 0:04:07 | | |  |
| 79 | Scientist | | 11/5/2017 | | 8,096 | | 31 | | 5 | | 0:03:27 | | |  |
| 79 | Reporter | | 11/5/2017 | | 5,561 | | 7 | | 7 | | 0:01:26 | | |  |
| 80 | Scientist | | 24/5/2017 | | 10,789 | | 18 | | 5 | | 0:02:50 | | |  |
| 80 | Reporter | | 24/5/2017 | | 4,236 | | 191 | | 18 | | 0:06:57 | | |  |
| 81 | Scientist | | 24/5/2017 | | 6,975 | | 79 | | 0 | | 0:02:51 | | |  |
| 81 | Reporter | | 22/5/2017 | | 3,424 | | 8 | | 2 | | 0:02:38 | | |  |
| 82 | Scientist | | 29/5/2017 | | 2,378 | | 1 | | 0 | | 0:03:10 | | |  |
| 82 | Reporter | | 29/5/2017 | | 32,271 | | 2,000 | | 8 | | 0:02:01 | | |  |
| 83 | Scientist | | 5/6/2017 | | 6,604 | | 38 | | 13 | | 0:03:28 | | |  |
| 83 | Reporter | | 5/6/2017 | | 24,727 | | 57 | | 21 | | 0:03:00 | | |  |
| 84 | Scientist | | 8/6/2017 | | 2,675 | | 18 | | 1 | | 0:00:59 | | |  |
| 84 | Reporter | | 7/6/2017 | | 11,076 | | 38 | | 8 | | 0:03:22 | | |  |
| 85 | Scientist | | 16/6/2017 | | 7,410 | | 43 | | 10 | | 0:04:39 | | |  |
| 85 | Reporter | | 18/6/2017 | | 91,325 | | 63 | | 27 | | 0:02:51 | | |  |
| 86 | Scientist | | 29/6/2017 | | 6,848 | | 97 | | 30 | | 0:03:39 | | |  |
| 86 | Reporter | | 26/6/2017 | | 2,412 | | 0 | | 1 | | 0:01:59 | | |  |
| 87 | Scientist | | 3/7/2017 | | 3,747 | | 23 | | 1 | | 0:01:23 | | |  |
| 87 | Reporter | | 4/7/2017 | | 23,592 | | 82 | | 6 | | 0:02:30 | | |  |
| 88 | Scientist | | 5/7/2017 | | 8,772 | | 13 | | 1 | | 0:02:15 | | |  |
| 88 | Reporter | | 5/7/2017 | | 19,475 | | 2 | | 7 | | 0:02:31 | | |  |
| 89 | Scientist | | 19/7/2017 | | 4,403 | | 29 | | 2 | | 0:02:13 | | |  |
| 89 | Reporter | | 19/7/2017 | | 7,703 | | 30 | | 37 | | 0:04:27 | | |  |
| 90 | Scientist | | 23/7/2017 | | 6,602 | | 6 | | 0 | | 0:03:13 | | |  |
| 90 | Reporter | | 23/7/2017 | | 13,323 | | 55 | | 5 | | 0:04:29 | | |  |
| 91 | Scientist | | 3/8/2017 | | 722 | | 1 | | 3 | | 0:00:54 | | |  |
| 91 | Reporter | | 3/8/2017 | | 10,600 | | 13 | | 3 | | 0:01:50 | | |  |
| 92 | Scientist | | 6/8/2017 | | 2,923 | | 2 | | 1 | | 0:01:09 | | |  |
| 92 | Reporter | | 9/8/2017 | | 9,076 | | 747 | | 10 | | 0:04:06 | | |  |
| 93 | Scientist | | 14/8/2017 | | 3,643 | | 0 | | 0 | | 0:01:13 | | |  |
| 93 | Reporter | | 15/8/2017 | | 5,511 | | 30 | | 3 | | 0:05:18 | | |  |
| 94 | Scientist | | 23/8/2017 | | 16,798 | | 19 | | 1 | | 0:04:11 | | |  |
| 94 | Reporter | | 21/8/2017 | | 5,515 | | 3 | | 2 | | 0:03:35 | | |  |
|  | |  | |  | |  | |  | |  | |  | | |
